# Convalescent Plasma Treatment in Patients with Covid-19: A Systematic Review and Meta-Analysis

**DOI:** 10.3389/fimmu.2022.817829

**Published:** 2022-02-07

**Authors:** Anselm Jorda, Manuel Kussmann, Nebu Kolenchery, Jolanta M. Siller-Matula, Markus Zeitlinger, Bernd Jilma, Georg Gelbenegger

**Affiliations:** ^1^ Department of Clinical Pharmacology, Medical University of Vienna, Vienna, Austria; ^2^ Division of Infectious Diseases and Tropical Medicine, Department of Medicine I, Medical University of Vienna, Vienna, Austria; ^3^ Department of Public Health, Saint Louis County, St. Louis, MO, United States; ^4^ Division of Cardiology, Department of Medicine II, Medical University of Vienna, Vienna, Austria; ^5^ Department of Experimental and Clinical Pharmacology, Center for Preclinical Research and Technology (CEPT), Medical University of Warsaw, Warsaw, Poland

**Keywords:** antibodies, passive immunization, SARS-CoV-2, convalescent plasma (CP) therapy, coronavirus – COVID-19, serotherapy, hyperimmune globulin

## Abstract

Convalescent plasma is a suggested treatment for Coronavirus disease 2019 (Covid-19), but its efficacy is uncertain. We aimed to evaluate whether the use of convalescent plasma is associated with improved clinical outcomes in patients with Covid-19.In this systematic review and meta-analysis, we searched randomized controlled trials investigating the use of convalescent plasma in patients with Covid-19 in Medline, Embase, Web of Science, Cochrane Library, and medRxiv from inception to October 17^th^, 2021. Two reviewers independently extracted the data. The primary efficacy outcome was all-cause mortality. The Cochrane Risk of Bias Tool and GRADE (Grading of Recommendations Assessment, Development and Evaluation) method were used. This study was registered with PROSPERO, CRD42021284861. Of the 8874 studies identified in the initial search, sixteen trials comprising 16 317 patients with Covid-19 were included. In the overall population, the all-cause mortality was 23.8% (2025 of 8524) with convalescent plasma and 24.4% (1903 of 7769) with standard of care (risk ratio (RR) 0.97, 95% CI 0.90-1.04) (high-certainty evidence). All-cause mortality did not differ in the subgroups of noncritically ill (21.7% [1288 of 5929] vs. 22.4% [1320 of 5882]) and critically ill (36.9% [518 of 1404] vs. 36.4% [455 of 1247]) patients with Covid-19. The use of convalescent plasma in patients who tested negative for anti-SARS-CoV-2 antibodies at baseline was not associated with significantly improved survival (RR 0.94, 95% CI 0.87-1.02). In the overall study population, initiation of mechanical ventilation (RR 0.97, 95% CI 0.88-1.07), time to clinical improvement (HR 1.09, 95% CI 0.91-1.30), and time to discharge (HR 0.95, 95% CI 0.89-1.02) were similar between the two groups. In patients with Covid-19, treatment with convalescent plasma, as compared with control, was not associated with lower all-cause mortality or improved disease progression, irrespective of disease severity and baseline antibody status.

**Systematic Review Registration:**

https://www.crd.york.ac.uk/prospero/, identifier PROSPERO (CRD42021284861).

## Introduction

Coronavirus disease 2019 (Covid-19) is an acute illness caused by the severe acute respiratory syndrome coronavirus 2 (SARS-CoV-2) that is associated with severe inflammation and organ dysfunction. Immunomodulatory treatments for Covid-19 remain elusive with only a few strategies (glucocorticoids and tocilizumab) showing a clear survival benefit. Therapeutic use of plasma from individuals who have recovered from Covid-19 has been hypothesized to show clinical benefits, particularly in immunocompromised patients and when used early in the course of the disease (1). The treatment rationale behind the use of convalescent plasma is to bridge the critical time period until a sufficient immune response is established in the infected patient (2). The use of convalescent plasma for the treatment of patients with Covid-19 has attracted widespread attention, yet definitive evidence of its efficacy is missing.

Observational data showed that convalescent plasma may have a role for patients who are immunocompromised and unable to adequately produce antibodies (3, 4). Further data suggested some benefits of targeting selected patient populations (non-intubated patients, age under 80 years) and using high-titer plasma (5–7). However, clinical data from randomized controlled trials were unable to reproduce these findings in an overall Covid-19 patient population.

With this systematic review we aimed to summarize all available data from published randomized controlled trials and discuss potential clinical implications. In the meta-analysis, we investigated whether convalescent plasma is associated with improved survival and disease progression.

## Methods

### Search Strategy and Selection Criteria

This meta-analysis has been reported in accordance with the Preferred Reporting Items for Systematic Review and Meta-analysis and performed according to established methods, as described previously (8). This meta-analysis was registered at PROSPERO under the registration number CRD42021284861. We employed a systematic search strategy in Medline, Embase, Web of Science, Cochrane Library and the preprint server medRxiv from database inception through October 17^th^, 2021 by searching for Covid-19 (and related terms) and convalescent plasma (and related terms) (9). The exact search strategies can be found in **Appendix Table 1**. Retrieved articles were assessed for their eligibility by reading the title and abstract and, if necessary, the full text. References of identified articles and previous meta-analysis or systematic reviews were searched for additional literature. There were no restrictions on language, publication date, publication status restrictions, or geographic region.

Only full-text articles were included in this meta-analysis. We included trials that (i) were randomized controlled trials (RCTs), (ii) compared convalescent plasma with standard of care or placebo, and (iii) reported on at least one of our outcomes of interest (all-cause mortality, requirement of mechanical ventilation, time to clinical improvement, time to hospital discharge). Ongoing, retrospective, other non-RCTs, and duplicate studies were excluded. Studies were excluded from the analysis if one could determine, from the title, abstract, or both that the study did not meet the inclusion criteria. If an article could not be excluded with certainty, the full text of the study in question was acquired and evaluated. The literature search and study selection were independently carried out by two reviewers (A.J. and G.G.). Any discrepancies were resolved with personal discussion and author consensus.

### Data Analysis

Selected trials included patients with Covid-19, that were being randomly allocated to convalescent plasma, standard-of-care treatment, or placebo and standard-of-care treatment. Randomized controlled trials were included regardless of the level of plasma titer (high or low antibody titer), number of patients included or healthcare setting (inpatient or outpatient). We extracted the following information for each RCT: trial design characteristics, number of patients included, patient demographics, convalescent plasma treatment details and regimen.

High antibody titer was defined as S-protein receptor-binding domain-specific IgG antibody titer of 1:640 or higher or serum neutralization titer of 1:40 or higher, according to previously used definitions (10).

The primary efficacy outcome was all-cause mortality. Secondary outcomes included requirement of mechanical ventilation after enrollment, time to clinical improvement, and time to hospital discharge. Due to variable endpoint definitions and study designs of the included trials, the pooling of other relevant endpoints was not feasible. We performed predefined subgroup analyses for all-cause mortality comparing critically ill and noncritically ill patients and patients with and without anti-SARS-CoV-2 antibodies at baseline. The definition of critically ill patients included those with shock or organ failure requiring admission to an intensive care unit (ICU), invasive mechanical ventilation, and/or vasopressors. Noncritically ill patients were those with moderate to severe Covid-19 not admitted to an ICU and without organ failure or shock. Sensitivity analyses were performed by removing each trial from the overall analyses and testing the impact of fixed- versus random-effect models of each outcome. Another sensitivity analysis involved the removal of preprint studies from the overall analysis. All reports eligible for analysis were assessed using the Cochrane Risk of Bias Tool. Publication bias was assessed by preparing funnel plots based on fixed-effect models of the key outcomes of the meta-analysis. Finally, the overall certainty of evidence for the primary and secondary outcomes was assessed according to the GRADE recommendations (11).

The data was extracted from full-text publications and, if available, supplementary files. Categorical variables are reported as frequencies and percentages. Results were pooled according to the inverse variance model. Risk ratios (RR) with 95% confidence intervals (95% CI) or hazard ratios (HR) with 95% CIs of each study and of pooled data are reported. Unadjusted p values are reported throughout, with hypothesis testing set at the two-tailed significance level of below 0.05. Heterogeneity between studies was assessed by inconsistency testing (I^2^). Percentages lower than 25% (I^2^ < 25%), between 25% and 50% (25% ≥ I^2^ < 50%), or 50% or higher (I^2^ ≥ 50%) correspond to low, medium and high heterogeneity, respectively. Due to high clinical heterogeneity of the included trials, a random-effect model was used. The statistical analysis was carried out using Review Manager (Version 5.4 Copenhagen: The Nordic Cochrane Centre, The Cochrane Collaboration, 2014).

### Role of Funding Source

There was no funding source for this study. G.G. is supported by a grant from the Austrian Science Funds (SFB54-P04) and by the Federal Ministry of Education, Science and Research for performing the ACOVACT trial.

## Results

**Figure 1:**
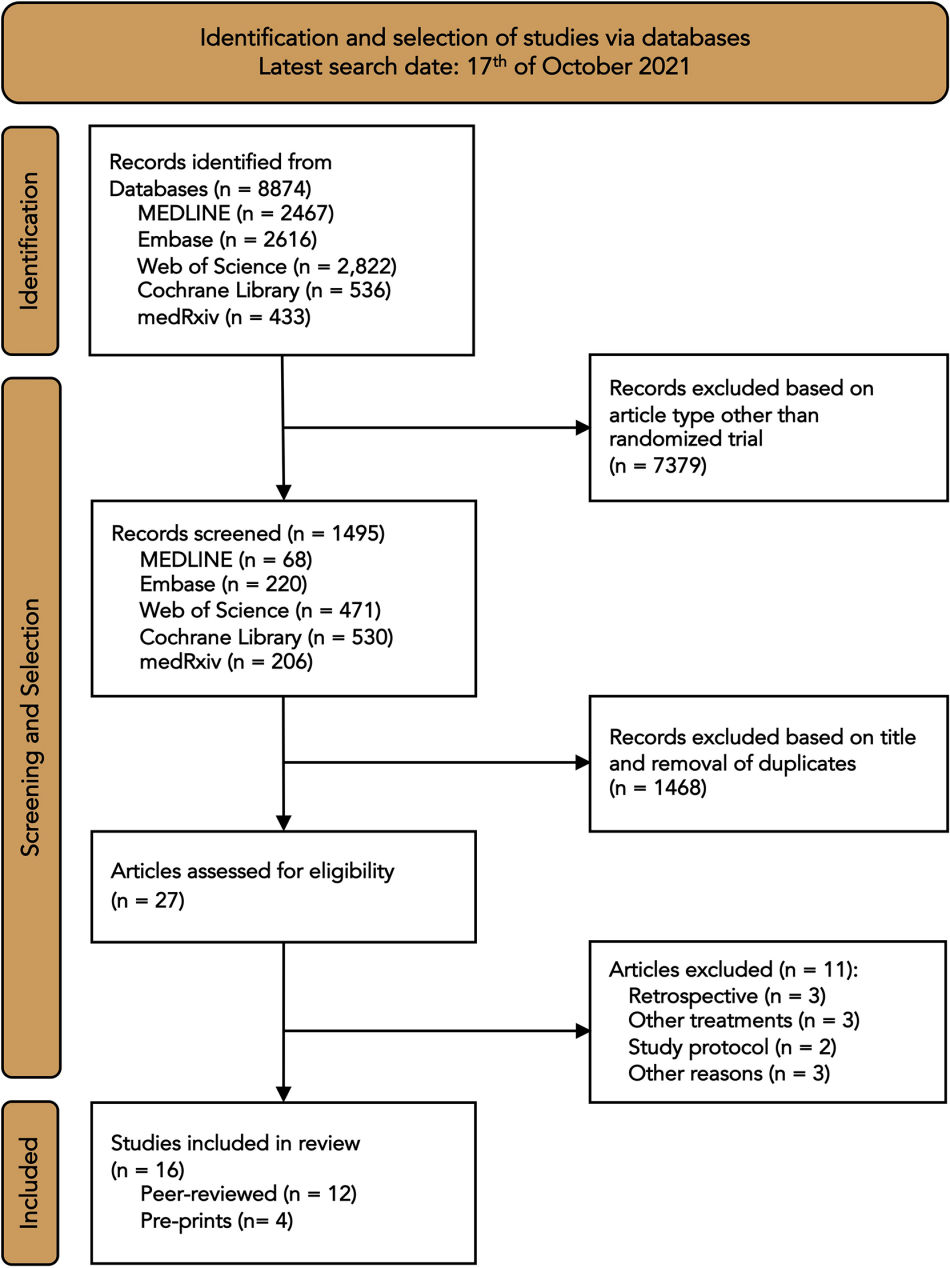
Flow diagram of search and selection process.

The literature search identified a total of 8874 records (**Figure 1**). After removal of duplicates and articles that were not randomized controlled trials, 27 articles were assessed for eligibility. Of these, eleven articles were excluded because they were retrospective studies (n=3), investigated other treatments (n=3), were study protocols (n=2), or because of other reasons (n=3). One trial was excluded because it was not a randomized trial (12). The final analysis included sixteen trials with a total of 16 317 patients. Twelve studies were published in peer-reviewed journals (13–24) and four were published on the preprint server medRxiv (25–28). Included trials were performed in North and South America, Europe, Asia and Australia (**Appendix Table 2**). Seven trials were terminated early, because of futility or poor recruitment. One trial was stopped early after emergency use authorization was granted for convalescent plasma in the United States (23). Four included trials were double-blind, placebo-controlled trials (20–23); one trial was single-blind (24), and the remaining trials were all open-label. The trials only included patients with confirmed Covid-19, except for the RECOVERY trial, which also included patients with suspected SARS-CoV-2 infection (18). Only one trial included outpatients (20). In one trial, patients were randomly allocated to either convalescent plasma or fresh frozen plasma in addition to the standard of care (26). Patients received a single infusion of convalescent plasma in eight trials and were given two infusions 24 hours apart in seven trials. Plasma antibody titers ranged from 1:100 to 1:1000. Five trials did not provide plasma titers (13, 14, 24, 26, 28). Eleven of the sixteen trials reported on the time from symptom onset to enrolment. Of these, nine trials had median durations from symptom onset to enrolment between 7 and 10 days (**Table 1**). The longest median duration was reported by the ChiCTR trial (median [IQR], 27 [22-39] vs.30 [19-38] days), and the shortest mean duration was reported by the INFANT-COVID-19 trial (mean ± SD, 1.7 ± 0.6 vs. 1.6 ± 0.6 days). Six trials assessed the anti-SARS-CoV-2 antibody status at baseline. In the convalescent plasma group, 56% of patients (3986 of 7120) had pre-existing antibodies and 33% (2417 of 7120) had no antibodies at baseline. In the control group, 52% of patients (3467 of 6690) had pre-existing antibodies and 29% (1992 of 6690) had no antibodies at baseline. The serologic status of the remaining patients was unknown.

**Table 1:** Characteristics of the included trials.

Study	Status	Illness severity	Symptom onset to enrolment (median days)	Blinding	Dose description	Titer	Control arm	N (n vs. n)
AlQahtani et al., 2021 (14)	Completed	Noncritical	Not reported	Open label	Two transfusions of 200 mL administered 24 h apart	Not specified	SOC	40 (20 vs. 20)
CONCOR-1, 2021 (15)	Terminated early	Noncritical and critical	8 (5–10) vs.8 (5–10)	Open label	Single transfusion of 500 mL	Low (>1:100 RBD)	SOC	921 (614 vs. 307)
ConCOVID, 2021 (17)	Terminated early	Noncritical and critical	9 (7–13) vs. 11 (6–16)	Open label	Single transfusion of 300 mL	Low (≥1:400 RBD)	SOC	86 (43 vs. 43)
ChiCTR, 2020 (19)	Terminated early	Noncritical and critical	27 (22-39) vs. 30 (19-38)	Open label	Single transfusion of 4 to 13 mL/kg body weight	High (not specified)	SOC	103 (52 vs. 51)
O’Donnell, 2021 (21)	Completed	Noncritical and critical	10 (7–13) vs. 9 (7–11)	Double-blind	Single transfusion of 200 to 250 mL	Low (1:400)	SOC + placebo	223 (150 vs. 73)
PLACID, 2020 (13)	Completed	Noncritical	8 (6-11) vs. 8 (6-11)	Open label	Two transfusions of 200 mL administered 24 h apart	Not specified	SOC	464 (235 vs. 229)
RECOVERY, 2021 (18)	Completed	Noncritical and critical	9 (6–12) vs. 9 (6–12)	Open label	Two transfusions of 200 to 350 mL administered 12 h apart	High (neutralizing titers of 1:100)	SOC	11 558 (5795 vs. 5763)
REMAP-CAP, 2021 (16)	Terminated according to protocol	Critical	Not reported	Open label	Two transfusions of 550 ± 150 mL	High (not specified)	SOC	2000 (1084 vs. 916)
PlasmAr, 2021 (22)	Completed	Noncritical and critical	8 (5–10) vs. 8 (5–10)	Double-blind	Single transfusion of 5 to 10 mL/kg body weight	High (≥1:800 RBD)	SOC + placebo	333 (228 vs. 105)
Bennett-Guerrero et al., 2021 (23)	Terminated early	Noncritical and critical	9 (6–18) vs. 9 (6–15)	Double-blind	Two transfusions of 480 mL	≥145 reflectance light units for IgG	SOC + placebo	74 (59 vs. 15)
Pouladzadeh et al., 2021 (24)	Completed	Noncritical and critical	Not reported	Single-blind	One or two transfusions of 500mL	Not specified	SOC	60 (30 vs. 30)
INFANT-COVID-19, 2021 (20)	Terminated early	Noncritical	1.7 ± 0.6 vs. 1.6 ± 0.6*	Double-blind	Single transfusion of 250mL	High (IgG titer > 1:1000)	SOC + placebo	160 (80 vs. 80)
ConPlas-19 (preprint) (25)	Terminated early	Noncritical	8 (7–9) vs. 8 (6-9)	Open label	Single transfusion of 250 to 300 mL	High (VMNT-ID50: all titers >1:80)	SOC	81 (38 vs. 43)
PICP19 (preprint) (28)	Not reported	Noncritical	Not reported	Open label	Two transfusions of 200mL	Not specified	SOC	80 (40 vs. 40)
CAPSID (preprint) (27)	Not reported	Noncritical and critical	7 (2-9) vs. 7 (5-10.5)	Open label	Three transfusions on day 1, 3, 5	Median PRNT50 titer 1:160 IQR: 1:80 to 1:320	SOC	105 (53 vs. 52)
ILBS-COVID-02 (preprint) (26)	Not reported	Noncritical and critical	Not reported	Open label	Two transfusions of 500 mL administered 24 h apart	Not specified	SOC + FFP	29 (14 vs. 15)

IgG, immunoglobulin G; PRNT50, concentration of serum to reduce the number of plaques by 50%; RBD, receptor-binding domain; SOC, standard of care; VMNT-ID50 virus microneutralization test - ID50% assay.

*mean ± standard deviation.

Using the Cochrane Risk of Bias Tool, the risk of bias of the key outcomes of this meta-analysis was assessed as low for most of the trials (**Appendix Table 3**). In two trials, some concerns were associated with the risk of bias arising from the randomization process (14, 25). The risk of bias was deemed high in one trial because of incomplete reporting on randomization and treatment allocation and adherence (28). Funnel plots did not show obvious asymmetry, indicating no clear evidence of publication bias (**Appendix Figure 1**).

The primary endpoint all-cause mortality was assessed in all sixteen trials. All-cause mortality was assessed from 15 to 30 days after randomization in fourteen trials. Two trials assessed all-cause mortality 60 days after randomization (17, 24), and one trial did not provide the length of follow-up (14). Five trials only included noncritically ill patients (13, 14, 20, 25, 28), and one trial included only critically ill patients with Covid-19 (16). Of the remaining ten trials, two trials provided subgroup analyses for all-cause mortality in noncritically and critically ill patients (18, 19). Two trials performed a subgroup analysis of all-cause mortality according to the anti-SARS-CoV-2 antibody status at baseline (16, 18).

Data on the use of mechanical ventilation were available in seven trials (six peer-reviewed and one preprint). Time to hospital discharge was assessed in eight trials, only one of which was published as a preprint. All trials reporting on time to hospital discharge provided hazard ratios. Similarly, four trials provided data on time to clinical improvement (three published in peer-reviewed journals, one as a preprint) using hazard ratios.

**Figure 2:**
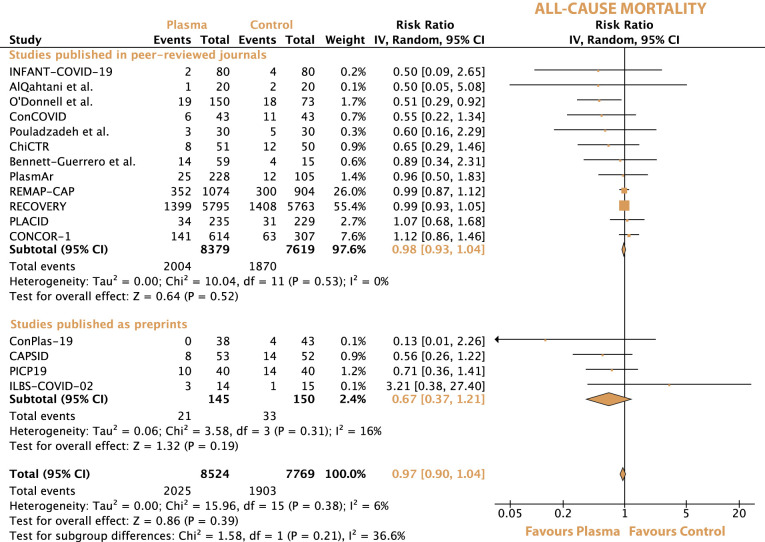
Forrest plot depicting the risk ratio of all-cause mortality between treatment with convalescent plasma and standard of care alone.

In the overall population, the all-cause mortality was 23.8% (2025 of 8524) with convalescent plasma and 24.4% (1903 of 7769) with standard of care. The risk ratio for all-cause mortality between convalescent plasma and standard of care was 0.97 (95% CI 0.90-1.04, p = 0.39) (**Figure 2**). After excluding the preprints, the all-cause mortality was 23.9% (2004 of 8379) with convalescent plasma and 24.5% (1870 of 7619) with standard of care alone, resulting in a risk ratio of 0.98 (95% CI 0.93-1.04, p = 0.53).

Convalescent plasma neither decreased the risk for all-cause mortality in noncritically ill patients (21.7% [1288 of 5929] vs. 22.4% [1320 of 5882]) nor in critically ill patients with Covid-19 (36.9% [518 of 1404] vs. 36,4% [455 of 1247]). The risk ratios for all-cause mortality were 0.97 (95% CI 0.91-1.04, p = 0.38) in noncritically ill patients and 1.04 (95% CI 0.93-1.16, p = 0.49) in critically ill patients (**Appendix Figure 2**).

All-cause mortality did not differ significantly in patients with or without preexisting anti-SARS-CoV-2 antibodies at baseline (20.8% [765 of 3675] vs. 19.8% [636 of 3219]) and 33.8% [772 of 2286] vs. 35.2% [636 of 1808]), respectively) (**Appendix Figure 3**). The respective risk ratios for all-cause mortality were 1.03 (95% CI 0.93-1.12, p = 0.6) in patients with preexisting anti-SARS-CoV-2 antibodies and 0.94 (95% CI 0.87-1.02, p = 0.16) in patients without antibodies.

Initiation of mechanical ventilation was required in 11.8% (734 of 6236) of patients with convalescent plasma and in 12.2% (734 of 5993) of patients with standard of care (RR 0.97, 95% CI 0.88-1.07, p = 0.54) (**Appendix Figure 4**).

The time to clinical improvement was reported by four trials. The definitions of clinical improvement varied among the trials and were specified as improvement of one or two points on similar but not identical ordinal outcome scales (**Appendix Table 4**). The median days to clinical improvement are provided in **Appendix Table 4**. Overall, the time to clinical improvement was similar between patients receiving convalescent plasma and the control group (HR 1.09, 95% CI 0.91-1.30, p = 0.37) (**Appendix Figure 5**).

Given the different levels of illness severity, the median time to hospital discharge varied considerably among the seven trials included in this analysis. The REMAP-CAP trial (16) reported the longest median time to hospital discharge between convalescent plasma and control (44 vs. 39 days, HR 0.95, 95% CI 0.86-1.06), and the trial by Pouladzadeh at al. (24). reported the shortest mean hospital stay (8.7 ± 3.9 vs. 6.7 ± 4.3 days, HR 0.37, 95% CI 0.02-6.84) (**Appendix Table 5**). Overall, the use of convalescent plasma, as compared with control, was not associated with a reduced time to hospital discharge (HR 0.95, 95% CI 0.89-1.02, p = 0.19) (**Appendix Figure 6**).

The sequential exclusion of each trial from the overall analyses did not change the pooled risk ratios and hazard ratios for any of the outcomes significantly. The exclusion of the preprints also did not change any of the pooled outcomes. For all-cause mortality, there was no statistically significant subgroup difference and a medium level of heterogeneity between peer-reviewed articles and preprints (Chi^2^ = 1.58, I^2^ = 36.6%, p = 0.21) (**Figure 2**). For all-cause mortality between noncritically and critically ill patients, there was no statistically significant subgroup difference and a low level of heterogeneity (Chi^2^ = 1.09, I^2^ = 8.2%, p = 0.3) (**Appendix Figure 2**). No statistically significant subgroup difference was observed between seronegative and seropositive patients in terms of all-cause mortality (Chi^2^ = 1.79, I^2^ = 44.1%, p = 0.18). Switching from a random-effect model to a fixed-effect model did not influence the outcomes of the meta-analyses significantly.

According to the GRADE assessment, the evidence for the observed effect of convalescent plasma on all-cause mortality is high (**Appendix Table 6**). The width of the 95% confidence interval (0.93-1.04 without preprints and 0.90-1.04 with preprints) makes substantial clinical effects on mortality unlikely in the given patient population. Further factors contributing to the high level of certainty of evidence include the large sample size (over 16 000 patients), the objective endpoint death, the low level of heterogeneity (I^2^ = 6%) and the robustness to sensitivity analyses. Similarly, the certainty of evidence for the use of mechanical ventilation was rated as high. The evidence for the effect of convalescent plasma on the time to hospital discharge was downgraded to moderate because of moderate concerns regarding the risk of bias, which might have been introduced by incomplete reporting and the subjectiveness of the endpoint. The evidence for the time of clinical improvement was downgraded to very low because of serious concerns regarding the risk of bias, incomplete reporting, heterogenous endpoint definitions, and imprecision (95% CI 0.91-1.30).

## Discussion

**Figure 3:**
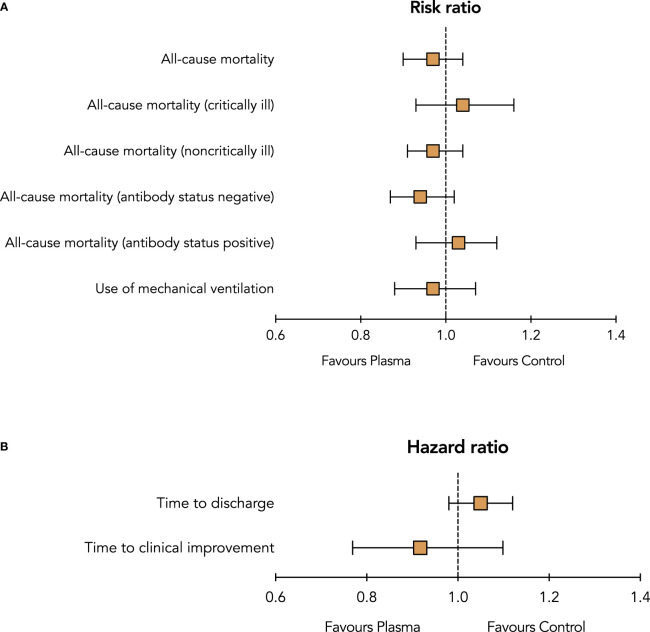
Summary risk ratios **(A)** and hazard ratios **(B)** of outcomes between treatment with convalescent plasma and control (standard of care with or without placebo).

In this meta-analysis that included sixteen RCTs with over 16 000 patients with Covid-19, there was no significant difference in all-cause mortality or any other clinical outcomes between treatment with convalescent plasma and control (standard of care alone or standard of care and placebo) (**Figure 3**). Similarly, there was no difference in all-cause mortality between convalescent plasma and control in the subgroups of critically ill or noncritically ill patients and in patients without anti-SARS-CoV-2 antibodies at baseline. This meta-analysis confirms the results of previous analyses which did not support the routine use of convalescent plasma.

So far, very few immunomodulatory agents, glucocorticoids and interleukin-6 antagonists, have been shown to significantly reduce mortality in patients hospitalized with Covid-19 (29, 30). Failure of RCTs to show a significant survival benefit of convalescent plasma could be due to a number of reasons: (i) In contrast to other pharmacological treatments against Covid-19, convalescent plasma is not artificially produced but collected from patients who recovered from a SARS-CoV-2 infection. Although the U.S. Food and Drug Administration provides guidance on the collection and use of convalescent plasma (31), it is inherently variable, which may confound the evidence of its potential benefits. In the sixteen included trials, six titer cut-offs using different SARS-CoV-2 antibody detection assays were defined, six trials did not specify any thresholds, and almost all trials administered different plasma volumes (**Table 1**); (ii) most patients were included more than seven days after symptom onset. Delayed patient inclusion might have concealed potential therapeutic effects of convalescent plasma; (iii) the type of SARS-CoV-2 variant of the infected individual may also affect the patient’s clinical response to treatment with convalescent plasma. SARS-CoV-2 variant types, of both the infected patient and the infused convalescent plasma, were not reported; (iv) cumulatively, more than 50% of patients in the treatment group tested positive for preexisting anti-SARS-CoV-2 antibodies at baseline, while around 30% of patients in the treatment group tested negative. Considering that the anticipated treatment effect of convalescent plasma is the highest in patients without adequate immune response, the vast inclusion of immunocompetent patients might have confounded the results. The question remains whether the absence of baseline anti-SARS-CoV-2 antibodies may potentially be helpful to guide the appropriate use of convalescent plasma. Our subgroup analysis, although possibly underpowered, showed no significant survival benefit of convalescent plasma over control in patients who tested negative for anti-SARS-CoV-2 antibodies at baseline.

In light of these uncertainties, it is unclear whether different plasma products, given at different stages of disease progression, may convey therapeutic benefits. The expected - but to this day undetected - treatment benefit might only apply for selected populations, such as immunocompromised patients. Clinical trials have included an overall patient population with Covid-19, irrespective of immunocompetency, and were therefore unable to determine the efficacy of convalescent plasma in immunocompromised patients. Treatment advantages of convalescent plasma have been observed in immuno-compromised patients (32, 33) but lack of data from prospective RCTs precludes clear recommendations for this particular patient population. One larger trial, although only of observational nature, investigating the efficacy and safety of convalescent plasma in immunocompromised patients is currently underway (NCT04884477).

No formal analysis was performed on the safety profile or serious adverse events because of limited data availability and inadequate quality of data. The use of convalescent plasma is deemed safe, with a low incidence of serious adverse events (5).

The RECOVERY trial was the only study that was powered for the primary endpoint all-cause mortality. The remaining trials were potentially susceptible to biased adjudication of primary and secondary outcomes (use of ventilation, time to clinical improvement, time to hospital discharge, clinical status, or disease progression) due to their open-label design (34).

The current guidelines from the National Institute of Health already recommend against the use of convalescent plasma in patients without impaired immunity but acknowledge insufficient evidence to recommend either for or against the use of convalescent plasma in immunocompromised patients with Covid-19 (35). Considering the overall lack of evidence for convalescent plasma in patients with Covid-19, the associated high treatment costs (36), and tenuous supply (especially when only high-titer plasma is sought) may contribute to a negative cost-effectiveness balance and may not warrant routine clinical use. In addition, the recent emergence of neutralizing monoclonal antibodies against SARS-CoV-2, having already shown a good clinical efficacy and safety profile, may render the use of convalescent plasma obsolete in the future (37–40).

The main strength of this meta-analysis is the large sample size of over 16 000 patients and the low heterogeneity of all-cause mortality among the trials. Considering the high quality of most of the included RCTs, the results of this meta-analysis provide a high certainty of evidence and should assist physician and health care providers in their decision-making in the current pandemic.

This study has several limitations. First, data from four studies were only available as preprint versions, which have not yet been peer-reviewed. However, they only contributed a small proportion of the patient population, and sensitivity analyses showed that the results were not changed by these preprints. Second, treatment regimens of convalescent plasma varied significantly between trials. Nine trials did not define the time window of symptom onset to treatment. Third, time of outcome assessment of the primary endpoint was not the same between trials. Fourth, twelve of the sixteen trials were open-label trials, which may have influenced the assessment of clinical outcomes. Fifth, contrary to the overall analysis, the subgroup analyses were possibly underpowered and should be interpreted with caution. Sixth, except for one trial (20), the results of this meta-analysis only apply to patients hospitalized with moderate or severe Covid-19. The efficacy of convalescent plasma in mild Covid-19 remains unclear. Seventh, the RECOVERY trial contributed to 71% of patients (11 558 of 16 293) and 55% of the weight of the meta-analysis in the random-effects model. Notably, the results of the RECOVERY trial were consistent with the pooled outcomes of the remaining studies. Eighth, trials did not provide sufficient data to assess the potential therapeutic benefit of convalescent plasma in patients with Covid-19 and impaired immunity or increased inflammatory markers.

In conclusion, convalescent plasma treatment compared with control was not associated with a significant decrease in all-cause mortality or with any improvement of other clinical outcomes in the overall patient population, consisting of critically ill and noncritically ill patients with Covid-19. Considering the high certainty of evidence, these results do not support the routine clinical use of convalescent plasma in patients with Covid-19.

## Data Availability Statement

The raw data supporting the conclusions of this article will be made available by the authors, without undue reservation.

## Author Contributions

GG conceived the idea. AJ and GG performed the research, interpreted the results, and drafted the manuscript. All authors critically revised the manuscript and approved the final version for publication.

## Conflict of Interest

The authors declare that the research was conducted in the absence of any commercial or financial relationships that could be construed as a potential conflict of interest.

## Publisher’s Note

All claims expressed in this article are solely those of the authors and do not necessarily represent those of their affiliated organizations, or those of the publisher, the editors and the reviewers. Any product that may be evaluated in this article, or claim that may be made by its manufacturer, is not guaranteed or endorsed by the publisher.
