# Supplementary material for: Convalescent Plasma Treatment in Patients with Covid-19: A Systematic Review and Meta-Analysis
Source: Front Immunol. 2022 Feb 7;13:817829. doi: 10.3389/fimmu.2022.817829 (PMC8859444; doi:10.3389/fimmu.2022.817829)
Supplement: Supplementary file 1 [file DataSheet_1.pdf]

# Supplementary Appendix

## Convalescent plasma treatment in patients with Covid-19: a systematic review and meta-analysis

Anselm Jorda, Manuel Kussmann, Nebu Kolenchery, Jolanta M. Siller-Matula, Markus Zeitlinger, Bernd Jilma, Georg Gelbenegger

# Table of content

**Appendix Tables** ... 3

**Appendix Table 1** Systematic search strategy ... 3

**Appendix Table 2** Study design of included trials ... 4

**Appendix Table 3** Risk of bias assessment according to The Cochrane Risk of Bias Tool ... 6

**Appendix Table 4** Results and definitions of time to clinical improvement ... 7

**Appendix Table 5** Time to hospital discharge in days ... 8

**Appendix Table 6** Assessment of level of certainty of evidence according to GRADE recommendations ... 9

**Appendix Figures** ... 10

**Appendix Figure 1** Funnel plots depicting the effects estimates of the outcomes of the trials. ... 10

**Appendix Figure 2** Forrest plot depicting the risk ratio of all-cause mortality between convalescent plasma and control in the subgroups of non-critically and critically ill patients. ... 11

**Appendix Figure 3** Forrest plot depicting the risk ratio of all-cause mortality between convalescent plasma and control in the subgroups of patients with and without preexisting anti-SARS-CoV-2 antibodies at baseline. ... 12

**Appendix Figure 4** Forrest plot depicting the risk ratio of requirement of mechanical ventilation after enrollment between convalescent plasma and control ... 13

**Appendix Figure 5** Forrest plot depicting the hazard ratio for the time to clinical improvement between convalescent plasma and control. ... 14

**Appendix Figure 6** Forrest plot depicting the hazard ratio for the time to hospital discharge between convalescent plasma and control. 15

# Appendix Tables

## **Appendix Table 1** Systematic search strategy

| **Pubmed** | **N=** |
|---|---|
| (("corona"[Title/Abstract] OR "covid"[Title/Abstract] OR "Covid-19"[Title/Abstract] OR "Covid19"[Title/Abstract] OR "sars"[Title/Abstract] OR "sars2"[Title/Abstract] OR "sars-2"[Title/Abstract] OR "sars-cov"[Title/Abstract] OR "sars-cov-2"[Title/Abstract] OR "severe acute respiratory syndrome"[Title/Abstract] OR "ncov"[Title/Abstract] OR "2019-ncov"[Title/Abstract]) AND ("convalescent"[Title/Abstract] OR "convalescence"[Title/Abstract] OR "hyperimmune plasma"[Title/Abstract] OR "immune plasma"[Title/Abstract] OR "passive immunization"[Title/Abstract] OR "plasma therapy"[Title/Abstract] OR "serotherapy"[Title/Abstract] OR "hyperimmune globulin therapy"[Title/Abstract] OR "passive antibody transfer"[Title/Abstract])) | **2,467** |
| (("corona"[Title/Abstract] OR "covid"[Title/Abstract] OR "Covid-19"[Title/Abstract] OR "Covid19"[Title/Abstract] OR "sars"[Title/Abstract] OR "sars2"[Title/Abstract] OR "sars-2"[Title/Abstract] OR "sars-cov"[Title/Abstract] OR "sars-cov-2"[Title/Abstract] OR "severe acute respiratory syndrome"[Title/Abstract] OR "ncov"[Title/Abstract] OR "2019-ncov"[Title/Abstract]) AND ("convalescent"[Title/Abstract] OR "convalescence"[Title/Abstract] OR "hyperimmune plasma"[Title/Abstract] OR "immune plasma"[Title/Abstract] OR "passive immunization"[Title/Abstract] OR "plasma therapy"[Title/Abstract] OR "serotherapy"[Title/Abstract] OR "hyperimmune globulin therapy"[Title/Abstract] OR "passive antibody transfer"[Title/Abstract])) AND (clinicaltrial [Filter]) | **68** |
| **Embase** | |
| ('corona':ab,ti OR 'covid':ab,ti OR 'covid-19':ab,ti OR 'covid19':ab,ti OR 'sars':ab,ti OR 'sars2':ab,ti OR 'sars-2':ab,ti OR 'sars-cov':ab,ti OR 'sars-cov-2':ab,ti OR 'severe acute respiratory syndrome':ab,ti OR 'ncov':ab,ti OR '2019-ncov':ab,ti) AND ('convalescent':ab,ti OR 'convalescence':ab,ti OR 'hyperimmune plasma':ab,ti OR 'immune plasma':ab,ti OR 'passive immunization':ab,ti OR 'plasma therapy':ab,ti OR 'serotherapy':ab,ti OR 'hyperimmune globulin therapy':ab,ti OR 'passive antibody transfer':ab,ti) | **2,616** |
| ('corona':ab,ti OR 'covid':ab,ti OR 'covid-19':ab,ti OR 'covid19':ab,ti OR 'sars':ab,ti OR 'sars2':ab,ti OR 'sars-2':ab,ti OR 'sars-cov':ab,ti OR 'sars-cov-2':ab,ti OR 'severe acute respiratory syndrome':ab,ti OR 'ncov':ab,ti OR '2019-ncov':ab,ti) AND ('convalescent':ab,ti OR 'convalescence':ab,ti OR 'hyperimmune plasma':ab,ti OR 'immune plasma':ab,ti OR 'passive immunization':ab,ti OR 'plasma therapy':ab,ti OR 'serotherapy':ab,ti OR 'hyperimmune globulin therapy':ab,ti OR 'passive antibody transfer':ab,ti) AND ('trial':ab,ti) | **220** |
| **Web of Science** | |
| (TI=(corona) OR AB=(corona) OR TI=(covid) OR AB=(covid) OR TI=( Covid-19) OR AB=( Covid-19) OR TI=( Covid19) OR AB=( Covid19) OR TI=(sars) OR AB=(sars) OR TI=(sarsa) OR AB=(sarsa) OR TI=(sars-2) OR AB=(sars-2) OR TI=(sars-cov) OR AB=(sars-cov) OR TI=(sars-cov-2) OR AB=(sars-cov-2) OR TI=(severe acute respiratory syndrome) OR AB=(severe acute respiratory syndrome) OR TI=(ncov) OR AB=(ncov) OR TI=(2019-ncov) OR AB=(2019-ncov)) AND (TI=(convalescent) OR AB=(convalescent) OR TI=( convalescence) OR AB=( convalescence) OR TI=(hyperimmune plasma) OR AB=(hyperimmune plasma) OR TI=(immune plasma) OR AB=(immune plasma) OR TI=(passive immunization) OR AB=(passive immunization) OR TI=(plasma therapy) OR AB=(plasma therapy) OR TI=(serotherapy) OR AB=(serotherapy) OR TI=(hyperimmune globulin therapy) OR AB=(hyperimmune globulin therapy) OR TI=(passive antibody transfer) OR AB=(passive antibody transfer)) | **2,822** |
| (TI=(corona) OR AB=(corona) OR TI=(covid) OR AB=(covid) OR TI=( Covid-19) OR AB=( Covid-19) OR TI=( Covid19) OR AB=( Covid19) OR TI=(sars) OR AB=(sars) OR TI=(sarsa) OR AB=(sarsa) OR TI=(sars-2) OR AB=(sars-2) OR TI=(sars-cov) OR AB=(sars-cov) OR TI=(sars-cov-2) OR AB=(sars-cov-2) OR TI=(severe acute respiratory syndrome) OR AB=(severe acute respiratory syndrome) OR TI=(ncov) OR AB=(ncov) OR TI=(2019-ncov) OR AB=(2019-ncov)) AND (TI=(convalescent) OR AB=(convalescent) OR TI=( convalescence) OR AB=( convalescence) OR TI=(hyperimmune plasma) OR AB=(hyperimmune plasma) OR TI=(immune plasma) OR AB=(immune plasma) OR TI=(passive immunization) OR AB=(passive immunization) OR TI=(plasma therapy) OR AB=(plasma therapy) OR TI=(serotherapy) OR AB=(serotherapy) OR TI=(hyperimmune globulin therapy) OR AB=(hyperimmune globulin therapy) OR TI=(passive antibody transfer) OR AB=(passive antibody transfer)) AND (TI=(trial) OR AB=(trial)) | **471** |
| **Cochrane Library** | |
| ((corona):ti,ab,kw OR (covid):ti,ab,kw OR (Covid-19):ti,ab,kw OR (Covid19):ti,ab,kw OR (sars):ti,ab,kw OR (sars2):ti,ab,kw OR (sars-2):ti,ab,kw OR (sars-cov):ti,ab,kw OR (sars-cov-2):ti,ab,kw OR (severe acute respiratory syndrome):ti,ab,kw OR (ncov):ti,ab,kw) AND ((convalescent):ti,ab,kw OR (convalescence):ti,ab,kw OR (hyperimmune plasma):ti,ab,kw OR (immune plasma):ti,ab,kw OR (passive immunization):ti,ab,kw OR (plasma therapy):ti,ab,kw OR (serotherapy):ti,ab,kw OR (hyperimmune globulin therapy):ti,ab,kw OR (passive antibody transfer):ti,ab,kw) | **536** |
| ((corona):ti,ab,kw OR (covid):ti,ab,kw OR (Covid-19):ti,ab,kw OR (Covid19):ti,ab,kw OR (sars):ti,ab,kw OR (sars2):ti,ab,kw OR (sars-2):ti,ab,kw OR (sars-cov):ti,ab,kw OR (sars-cov-2):ti,ab,kw OR (severe acute respiratory syndrome):ti,ab,kw OR (ncov):ti,ab,kw) AND ((convalescent):ti,ab,kw OR (convalescence):ti,ab,kw OR (hyperimmune plasma):ti,ab,kw OR (immune plasma):ti,ab,kw OR (passive immunization):ti,ab,kw OR (plasma therapy):ti,ab,kw OR (serotherapy):ti,ab,kw OR (hyperimmune globulin therapy):ti,ab,kw OR (passive antibody transfer):ti,ab,kw) AND TRIALS | **530** |
| **MedRxiv** | |
| ((covid*) OR (corona*) OR (sars*)) AND (convalescen*) | **433** |
| ((covid*) OR (corona*) OR (sars*)) AND (convalescen*) AND ((randomized controlled trial) OR (RCT)) | **206** |

# **Appendix Table 2** Study design of included trials

| Study | Countries | Design | Primary Efficacy Outcome Parameters | Secondary Efficacy Outcome Parameters | Safety Outcome Parameters | Inclusion criteria | Exclusion criteria | Symptom onset before enrollment |
|---|---|---|---|---|---|---|---|---|
| REMAP-CAP | Australia, Canada, United Kingdom, and United States | open-label, adaptive RCT | respiratory and cardiovascular organ support-free days up to day 21 | (1) in-hospital survival (2) 28-day survival (3) 90-day survival (4) respiratory support-free days (5) cardiovascular support-free days (6) progression to invasive mechanical ventilation, ECMO or death (8) ICU length of stay (9) hospital length of stay (10) WHO ordinal scale score at day 14 (11) venous thromboembolic events at 90 days (12) serious adverse events | not reported | (1) adult patients (2) admitted to hospital with acute illness due to confirmed Covid-19 | (1) death is deemed to be imminent and inevitable during the next 24 hours AND one or more of the patients, substitute decision maker or attending physician are not committed to full active treatment (2) patient is expected to be discharged from hospital today or tomorrow (3) more than 14 days have elapsed while admitted to hospital with symptoms of an acute illness due to suspected or proven pandemic infection (4) previous participation in this REMAP within the last 90 days | n.a. |
| CONCOR-1 | Canada, United States and Brazil | multicenter, open-label, randomized controlled trial | composite of intubation or death by day 30 | (1) time to intubation or death (2) ventilator-free days by day 30 (3) in-hospital death by day 90 (4) time to in-hospital death (5) death by day 30 (6) length of stay in critical care and hospital (7) need for extracorporeal membrane oxygenation (8) need for renal replacement therapy (9) convalescent plasma-associated adverse events (10) occurrence of ≥3 grade adverse events by day 30 | not reported | (1) ≥16 years of age in Canada or ≥18 years of age in the United States and Brazil (2) admitted to the hospital ward with confirmed COVID-19 (3) required supplemental oxygen (4) 500-ml of ABO-compatible COVID-19 convalescent plasma (CCP) was available | (1) more than 12 d from the onset of respiratory symptoms (2) imminent or current intubation (3) a contraindication to plasma transfusion (4) a plan for no active treatment | ≤ 12 d |
| O'Donnell | United States and Brazil | randomized, double-blind, controlled trial | clinical status at day | (1) time-to-clinical improvement (2) in-hospital mortality (3) 28-day mortality (4) time to discontinuation of supplemental oxygen (5) time to hospital discharge (6) serious and grade 3 and 4 adverse events | not reported | (1) hospitalized patients aged 18 years or older with evidence of SARS-CoV-2 infection by PCR of nasopharyngeal, oropharyngeal swab or tracheal aspirate sample within 14 days of randomization (2) infiltrates on chest imaging (3) oxygen saturation less than or equal to 94% on room air or requirement for supplemental oxygen, IMV, or ECMO | (1) receipt of any antiviral agent with possible activity against SARS-CoV-2 within 24 hours of randomization (2) duration of IMV or ECMO 5 days or longer at time of screening (3) severe multiorgan failure (4) history of prior reactions to transfusion blood products | n.a. |
| Bennett-Guerrero et al. | United States | randomized, double-blind, placebo-controlled trial | ventilator-free days from randomization to day 28 | (1) all-cause mortality through 90 days (2) WHO ordinal scale on each day through 28 days (3) immune response | not reported | (1) adult patients (2) hospitalized with a confirmed diagnosis of COVID-19 infection | (1) contraindication to transfusion or history of prior reactions to transfusion blood products (2) receipt of pooled (polyclonal) immunoglobulin or any intravenous polyclonal immunoglobulin in past 30 days (3) female subjects with positive pregnancy test, breastfeeding, or planning to become pregnant/breastfeed during the study period (4) in the treating physician's opinion, the patient is unable to tolerate a 450-550 mL infusion of plasma over up to 8 hours (4 hours max per unit) (5) unable to be randomized within 14 days of admission to Stony Brook Hospital | n.a. |
| RECOVERY | United Kingdom | open-label RCT | all-cause mortality | (1) time to discharge from hospital (2) receipt of invasive mechanical ventilation (3) death (4) receipt of ventilation (5) time to successful cessation of IMV (6) use of renal dialysis or haemofiltration | (1) transfusion related adverse events at 72 h following randomisation (2) cause-specific mortality (3) major cardiac arrhythmia | (1) hospitalized (2) SARS-Cov-2 infection (3) no medical history that might, in the opinion of the attending clinician, put the patient at significant risk | (1) drug not available | n.a. |
| ConCOVID | Netherlands | open-label, multicenter RCT | all-cause mortality until discharge from hospital or a maximum of 60 days after admission | (1) improvement on the eight-point WHO COVID-19 disease severity scale on day 15 and day 30 (2) hospital length of stay (3) SARS-CoV-2 shedding from the airways (4) impact of ConvP on humoral immunity and inflammation | (1) plasma-related transfusion reaction (2) death | (1) ≥ 18 years (2) admitted to the hospital for COVID-19 proven by a SARS-CoV-2 genome detectable in a RT-PCR test in the previous 96 h | (1) documented IgA deficiency (2) on mechanical ventilation for >96 h at the time of screening | n.a. |
| AlQahtani et al. | Bahrain | open-label RCT | requirement for NIV or MV | (1) C-reactive protein (2) procalcitonin (3) lactate dehydrogenase (4) troponin (5) ferritin (6) D-Dimer (7) brain natriuretic peptide (8) lactate changes (9) 28-day mortality rate | not reported | (1) signed informed consent (2) aged at least 21 years (3) COVID-19 diagnosis based on PCR testing (5) hypoxia (oxygen saturation of less than or equal 92% on air, or PO2 < 60 mmHg arterial blood gas, or arterial partial pressure of oxygen/fraction of inspired oxygen of 300 or less and the patient requiring oxygen therapy (6) pneumonia confirmed by chest imaging. | (1) Patients with mild disease not requiring oxygen therapy (2) Patients with a normal CXR or CT scan (3) Patients requiring ventilatory support (non-invasive or mechanical) (4) Patients with a negative PCR test for SARS-CoV-2 (5) Patients with a history of allergy to plasma, sodium citrate or methylene blue, or those with a history of autoimmune disease or selective IgA deficiency | ≤ 14 d |
| Pouladzadeh et al | Iran | single-blind, parallel-group RCT | levels of cytokine storm indices | (1) length of in-hospital stay (2) 2-month mortality after admission (3) improvement in the 8-point WHO severity score (4) frequency of CP therapy-related side effects | not reported | (1) COVID-19 patients who had specified COVID-19 symptoms (2) positive results of PCR test and CT scan (3) severity WHO score > 4 (4) oxygen saturation ≤ 93% in room air | not reported | ≤7d |
| PlasmAr | Argentina | double-blind, placebo-controlled, multicenter RCT | clinical status 30 days after intervention | (1) clinical status on the ordinal scale at days 7 and 14 (2) time (in days) to discharge from the hospital (3) time to discharge from the ICU (4) time to improvement in at least two categories on the ordinal scale (5) time to death (6) time to full functional recovery | not reported | (1) hospitalized adults (2) positve RT-PCR assay of a respiratory tract sample (3) radiologically confirmed pneumonia (4) no previous directives rejecting advanced life support (5) at least one of the following severity criteria: oxygen saturation <93% while they were at rest and breathing ambient air, a ratio of the partial pressure of oxygen (PaO2) to the fraction of inspired oxygen (FiO2) below 300 mm Hg (PaO2:FiO2), or a Sequential Organ Failure Assessment (SOFA) or modified SOFA score of two or more points above baseline status | (1) pregnant or lactating (2) patients of reproductive age who were not willing to use contraceptive measures for a period of 30 days after enrollment (3) patients with a history of blood component allergies, an infectious cause of pneumonia other than SARS-CoV-2, a requirement for mechanical ventilation, multiorgan failure, or any other condition that would impede the provision of informed consent | n.a. |
| INFANT-COVID-19 | Argentina | double-blind, placebo-controlled RCT | development of severe respiratory disease | (1) life-threatening respiratory disease (2) critical systemic illness (3) death associated with Covid-19 | not reported | (1) 75 years of age or older or between 65 and 74 years of age with at least one coexisting condition (hypertension or diabetes for which the patient was currently receiving pharmacologic treatment, obesity, chronic renal failure, cardiovascular disease, and COPD) (2) at the time of screening for SARS-CoV-2 by RT-PCR assay, eligible patients had had at least one of each sign or symptom in the following two categories for less than 48 hours: a temperature of at least 37.5°C, unexplained sweating, or chills; and dry cough, dyspnea, fatigue, myalgia, anorexia, sore throat, dysgeusia, anosmia, or rhinorrhea. | not reported | ≤ 72 h |

| | | | | | | | | |
|---|---|---|---|---|---|---|---|---|
| PLACID | India | open label, parallel-arm, phase II, multicentre, RCT | composite of progression to severe disease (PaO2/FiO2 ratio <100 mm Hg) within 28 days or all-cause mortality at 28 days | (1) time to symptom resolution (2) change in oxygen requirement after plasma transfusion (3) total duration of respiratory support during hospital admission (4) post-enrolment duration of respiratory support until day 28 or discharge (5) proportion of participants requiring invasive or non-invasive ventilation (6) sequential organ failure assessment score over days 0, 3, and 7 (7) conversion to a negative result for SARS-CoV-2 RNA on days 3 and 7 (8) levels of biomarkers (9)requirement of vasopressor support | (1) frequency of minor and serious adverse event within six hours of convalescent plasma transfusion | (1) moderate illness with either a partial pressure of oxygen in arterial blood/fraction of inspired oxygen (PaO2/FiO2) ratio between 200 mm Hg and 300 mm Hg or a respiratory rate of more than 24/min with oxygen saturation 93% or less on room air (2) availability of a matched donor for convalescent plasma at the point of enrolment | (1) pregnant and lactating women (2) known hypersensitivity to blood products (3) recipients of immunoglobulin in the past 30 days (4) conditions precluding infusion of blood products (5) participants in any other clinical trials (6) critically ill patients with PaO2/ FiO2 <200 mm Hg or shock (requiring vasopressors to maintain a mean arterial pressure (MAP) of ≥65 mm Hg or MAP of <65 mm Hg). | n.a. |
| ChiCTR | China | open-label, multicenter RCT | time to clinical improvement within a 28-day period | (1) 28-day mortality (2) duration of hospitalization (3) conversion of nasopharyngeal swab viral PCR results from positive at baseline to negative at follow-up assessed at 24, 48, and 72 hours | not reported | (1) signed informed consent (2) aged at least 18 years (3) COVID-19 diagnosis based on polymerase chain reaction (PCR) testing (4) positive PCR result within 72 hours prior to randomization (5) pneumonia confirmed by chest imaging (6) clinical symptoms meeting the definitions of severe or life-threatening COVID-19 (7) acceptance of random group assignment (8) hospital admission (9) willingness to participate in all necessary research studies and be able to complete the study follow-up (10) no participation in other clinical trials during the study period | (1) pregnancy or lactation (2) immunoglobulin allergy (3) IgA deficiency (4) preexisting comorbidity that could increase the risk of thrombosis (5) life expectancy less than 24 hours (6) disseminated intravascular coagulation (7) severe septic shock (8) PaO2/ FIO2 of less than 100 (9) severe congestive heart failure (10) detection of high titer of S protein–RBD-specific (receptor binding domain) IgG antibody (≥1:640) (11) other contraindications as determined by the patient's physicians (12) participation in any antiviral clinical trials for COVID-19 within 30 days | n.a. |
| CAPSID | Germany | open-label, multicenter RCT | dichotomous composite outcome of survival and no longer requiring ventilatory support or ICU treatment or tachypnea on day 21 | (1) time to clinical improvement | (1) frequency and severity of adverse events | (1) SARS-CoV-2 infection confirmed by PCR (bronchoalveolar lavage, sputum, nasal and/or pharyngeal swap) (2) age ≥ 18 years and ≤ 75 years (3) severe disease defined by at least one of the following: a) respiratory rate ≥ 30 breaths / minute under ambient air; b) requirement of any type of ventilation support; or c) needs treatment on ICU (4) written informed consent by patient or representative | (1) accompanying diseases other than COVID-19 with an expected survival time of less than 12 months (2) previous treatment with any SARS-CoV-2-convalescent plasma (3) in the opinion of the clinical team, progression to death is imminent and inevitable within the next 48 hours, irrespective of the provision of treatment (4) Interval > 72 hours since start of mechanical ventilation (5) not considered eligible for extracorporeal oxygenation support (6) chronic obstructive lung disease, stage 4 (7) lung fibrosis with usual interstitial pneumonia pattern in CT and severe emphysema (8) chronic heart failure NYHA ≥ 3 and/or pre-existing reduction of left ventricular ejection fraction to ≤ 30% (9) shock of any type requiring ≥ 0.5 μg/kg/min noradrenaline (or equivalent) or requiring more than two types of vasopressor medication for more than 8 hours (10) liver cirrhosis Child C (11) liver failure: bilirubin > 5 x upper limit of normal (ULN) and elevation of ALT /AST (at least one >10 x ULN); (12) any history of adverse reactions to plasma proteins (13) known deficiency of immunoglobulin A (14) pregnancy (15) breastfeeding women (16) volume overload until sufficiently treated (17) participation in another clinical trial with an investigational medicinal product | n.a. |
| PICP19 | India | open-label, single-center, phase II, RCT | all-cause mortality | not reported | not reported | (1) hospitalized patients with Covid-19 (2) oxygen supplementation | not reported | ≤ 14 d |
| ILBS-COVID-02 | India | open-label, single-center, phase II, RCT | proportion of patients remaining free of mechanical ventilation | not reported | not reported | (1) SARS-CoV-2 infection (positive by real-time PCR assay) patient (2) severe COVID-19 (respiratory rate (RR) 30/min, oxygen saturation level less than 93% in resting state, the partial pressure of oxygen (PaO2)/oxygen concentration (FiO2) 300 mmHg, lung infiltrates >50% within 24 to 48 hours]) | (1) failure to obtain informed consent (2) patients less than 18 years or more than 65 years of age (3) co-morbid conditions (cardiopulmonary disease-structural or valvular heart disease, coronary artery disease, COPD, chronic liver disease, chronic kidney disease) (4) multi-organ failure or on mechanical ventilation (5) pregnant females (6) HIV (7) viral hepatitis (8) cancer (9) morbid obesity with a BMI>35 kg/m2 (10) extremely moribund patients with an expected life expectancy of <24 hours (11) hemodynamic instability requiring vasopressors (12) previously known history of allergy to plasma (13) PaO2/FiO2 ratio less than 150 | ≤ 3 d |
| ConPlas-19 | Spain | open-label, multicenter RCT | proportion of patients in categories 5, 6 or 7 of the COVID-19 ordinal scale at day 15 | (1) time to clinical improvement (2) mean change in the ordinal scale from baseline (3) proportion of patients in categories 5, 6 or 7 at day 29 (4) mortality at days 15 and 29 (5) duration of hospital stay (6) number of days alive and free from oxygen support (7) number of days alive and free from MV | not reported | (1) hospitalized for laboratory-confirmed SARS-CoV-2 infection (RT-PCR) with either radiographic evidence of pulmonary infiltrates or clinical evidence plus SpO2 94% on room air (2) within 12 days from the onset of symptoms (fever or cough) | (1) excluded if already on mechanical ventilation (invasive or non-invasive) or high flow oxygen devices | ≤ 12 d |

Abbreviations: ECMO extracorporeal membrane oxygenation, ICU intensive care unit, MV mechanical ventilation, NIV non-invasive ventilation, RCT randomized controlled trial

**Appendix Table 3** Risk of bias assessment according to The Cochrane Risk of Bias Tool

| **Study** | Risk of selection bias (random sequence generation) | Risk of selection bias (allocation concealment) | Risk of performance bias | Risk of detection bias | Risk of bias attrition | Risk of reporting bias | **Overall risk of bias** |
|---|---|---|---|---|---|---|---|
| **AlQahtani et al., 2021** | Some | Low | Low | Low | Low | Low | **Some concerns** |
| **CONCOR-1, 2021** | Low | Low | Low | Low | Low | Low | **Low risk** |
| **ConCOVID, 2021** | Low | Low | Low | Low | Low | Low | **Low risk** |
| **ChiCTR, 2020** | Low | Low | Low | Low | Low | Low | **Low risk** |
| **O'Donnell, 2021** | Low | Low | Low | Low | Low | Low | **Low risk** |
| **PLACID, 2020** | Low | Low | Low | Low | Low | Low | **Low risk** |
| **RECOVERY, 2021** | Low | Low | Low | Low | Low | Low | **Low risk** |
| **REMAP-CAP, 2021** | Low | Low | Low | Low | Low | Low | **Low risk** |
| **PlasmAr, 2021** | Low | Low | Low | Low | Low | Low | **Low risk** |
| **Bennett-Guerrero, 2021** | Low | Low | Low | Low | Low | Low | **Low risk** |
| **Pouladzadeh et al., 2021** | Low | Low | Low | Low | Low | Low | **Low risk** |
| **INFANT-COVID-19, 2021** | Low | Low | Low | Low | Low | Low | **Low risk** |
| **ConPlas-19** | Some | Low | Low | Low | Low | Low | **Some concerns** |
| **PICP19** | Some | Some | Low | Low | Low | Low | **High risk** |
| **CAPSID** | Low | Low | Low | Low | Low | Low | **Low risk** |
| **ILBS-COVID-02** | Low | Low | Low | Low | Low | Low | **Low risk** |

**Appendix Table 4** Results and definitions of time to clinical improvement

| Study | Convalescent plasma | | Control | | HR (95% CI) | Definition of clinical improvement | Scale |
|---|---|---|---|---|---|---|---|
| | Median [IQR] days to clinical improvement | n= | Median [IQR] days to clinical improvement | n= | | | |
| O'Donnell, 2021 | 5 (4-6) | 150 | 7 (5-8) | 73 | 1.20 (0.87–1.64) | Discharge or improvement by 1 point on ordinal outcome scale | WHO scale:<br>1: not hospitalized with resumption of normal activities<br>2: not hospitalized, but unable to resume normal activities<br>3: hospitalized, not requiring supplemental oxygen<br>4: hospitalized, requiring supplemental oxygen<br>5: hospitalized, requiring high-flow oxygen therapy or NIMV<br>6: hospitalized, requiring ECMO, IMV<br>7: death |
| PlasmAr, 2021 | 12 (7-29) | 228 | 12 (6-ND) | 105 | 1.0 (0.76-1.32) | Discharge or improvement by 2 points in ordinal outcome scale | WHO scale (adapted)<br>1: indicated death<br>2: IMV<br>3: hospitalized with supplemental oxygen requirement,<br>4: hospitalized without supplemental oxygen requirement,<br>5: discharged without full return to baseline physical function<br>6: discharged with full return to baseline physical function |
| ChiCTR, 2020 | 28 (13-ND) | 52 | ND (18-ND) | 51 | 1.40 (0.79-2.49) | Discharge or improvement by 2 points on ordinal outcome scale | 1: hospital discharge.<br>2: hospitalization with no supplemental oxygen<br>3: hospitalization plus supplemental oxygen<br>4: hospitalization plus NIV or high-flow supplemental oxygen<br>5: hospitalization plus ECMO or IMV<br>6: death |
| ConPlas-19, preprint | 6.5 (4-9)* | 38 | 6 (5-8)* | 43 | 0.94 (0.59-1.50) | Improvement by 1 point on ordinal outcome scale | WHO scale:<br>1: not hospitalized with resumption of normal activities<br>2: not hospitalized, but unable to resume normal activities<br>3: hospitalized, not requiring supplemental oxygen<br>4: hospitalized, requiring supplemental oxygen<br>5: hospitalized, requiring high-flow oxygen therapy or NIMV<br>6: hospitalized, requiring ECMO, IMV<br>7: death |

*median (95% confidence interval)

Abbreviations: ECMO extracorporeal membrane oxygenation, IMV invasive mechanical ventilation, ND not determined, NIMV non-invasive mechanical ventilation

**Appendix Table 5** Time to hospital discharge in days

| Study | Convalescent plasma | | Control | | HR (95% CI) |
|---|---|---|---|---|---|
| | Days to discharge median (IQR) | n= | Days to discharge median (IQR) | n= | |
| CONCOR-1, 2021 | not reported | 625 | not reported | 313 | 0.91 (0.8-1.04) |
| ConCOVID, 2021 | not reported | 43 | not reported | 43 | 0.88 (0.49–1.60) |
| ChiCTR, 2020 | 28 (13-ND) | 52 | ND (19-ND) | 51 | 1.61 (0.88-2.95) |
| O'Donnell, 2021 | 9 (6-28) | 150 | 8 (6-22) | 73 | 1.02 (0.75-1.38) |
| REMAP-CAP, 2021 | 44 (not reported) | 1075 | 39 (not reported) | 905 | 0.95 (0.86-1.06) |
| PlasmAr, 2021 | 13 (8-30) | 228 | 12 (7-ND) | 105 | 1.0 (0.76-1.32) |
| Pouladzadeh, 2021 | 8.7 ± 3.9* | 30 | 6.7 ± 4.3* | 30 | 0.37 (0.02-6.84) |
| ConPlas-19 | 8.5 (6-13)† | 38 | 9 (6-11)† | 43 | 1.14 (0.71-1.81) |

*mean ± standard deviation

†median (95% confidence interval)

Abbreviation: ND not determined

**Appendix Table 6** Assessment of level of certainty of evidence according to GRADE recommendations

| Outcome | | All-cause mortality | Mechanical ventilation use | Time to clinical improvement | Time to discharge |
|---|---|---|---|---|---|
| Trials | | 16 RCTs mainly open label | 7 RCTs mainly open label | 4 RCTs mainly open label | 8 RCTs mainly open label |
| Number of patients | | 16,293 (8524 vs. 7769) | 12,229 (6236 vs. 5993) | 740 (468 vs. 272) | 3,807 (2239 vs. 1568) |
| Pooled effect (95% CI) | | RR 0.97 (0.90-1.04) | RR 0.97 (0.88-1.07) | HR 1.09 (0.91-1.30) | HR 0.95 (0.89-1.02) |
| Down-grading factors | Risk of bias | low | low | high | moderate |
| | Imprecision | low | low | high | low |
| | Inconsistency | low | low | low | low |
| | Indirectness | very low | low | moderate | low |
| | Publication bias | low | low | low | low |
| Up-grading factors | Large magnitude of effect | no | no | no | no |
| | Dose-response gradient | no | no | no | no |
| | All residual confounding would decrease magnitude of effect | n.a. | n.a. | n.a. | n.a. |
| **Level of certainty** | | **High** | **High** | **Very low** | **Moderate** |

## Appendix Figures

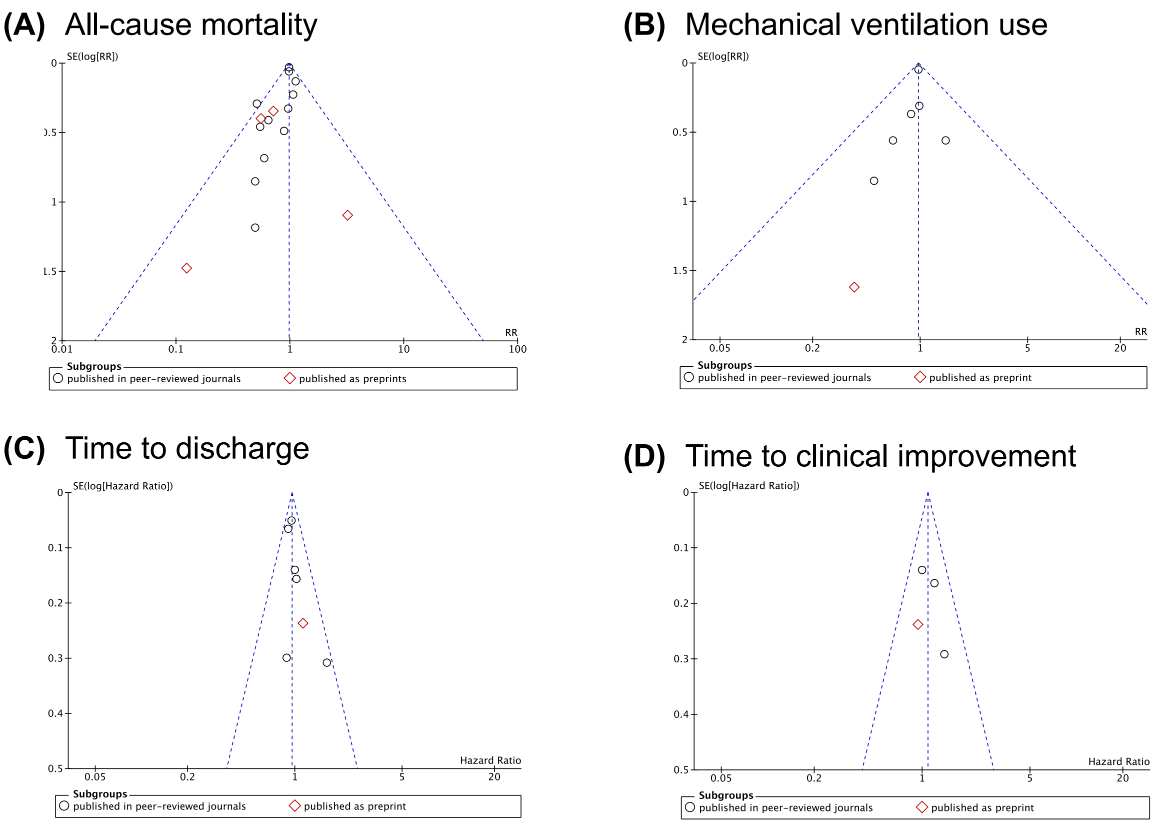


**Appendix Figure 1** Funnel plots depicting the effects estimates of the outcomes of the trials.

| Study or Subgroup | Plasma Events | Plasma Total | Control Events | Control Total | Weight | Risk Ratio IV, Random, 95% CI |
|---|---|---|---|---|---|---|
| **Non-critically ill patients** | | | | | | |
| ConPlas-19 | 0 | 38 | 4 | 43 | 0.0% | 0.13 [0.01, 2.26] |
| ChiCTR | 0 | 23 | 2 | 22 | 0.0% | 0.19 [0.01, 3.78] |
| INFANT-COVID-19 | 2 | 80 | 4 | 80 | 0.1% | 0.50 [0.09, 2.65] |
| AlQahtani et al. | 1 | 20 | 2 | 20 | 0.1% | 0.50 [0.05, 5.08] |
| PICP19 | 10 | 40 | 14 | 40 | 0.7% | 0.71 [0.36, 1.41] |
| RECOVERY | 1241 | 5493 | 1263 | 5448 | 65.5% | 0.97 [0.91, 1.04] |
| PLACID | 34 | 235 | 31 | 229 | 1.5% | 1.07 [0.68, 1.68] |
| **Subtotal (95% CI)** | | **5929** | | **5882** | **68.0%** | **0.97 [0.91, 1.04]** |
| Total events | 1288 | | 1320 | | | |
| Heterogeneity: $Tau^2 = 0.00$; $Chi^2 = 4.95$, df = 6 (P = 0.55); $I^2 = 0\%$ | | | | | | |
| Test for overall effect: Z = 0.88 (P = 0.38) | | | | | | |
| **Critically ill patients** | | | | | | |
| ChiCTR | 8 | 28 | 10 | 28 | 0.5% | 0.80 [0.37, 1.72] |
| REMAP-CAP | 352 | 1074 | 300 | 904 | 19.5% | 0.99 [0.87, 1.12] |
| RECOVERY | 158 | 302 | 145 | 315 | 12.0% | 1.14 [0.97, 1.33] |
| **Subtotal (95% CI)** | | **1404** | | **1247** | **32.0%** | **1.04 [0.93, 1.16]** |
| Total events | 518 | | 455 | | | |
| Heterogeneity: $Tau^2 = 0.00$; $Chi^2 = 2.26$, df = 2 (P = 0.32); $I^2 = 12\%$ | | | | | | |
| Test for overall effect: Z = 0.69 (P = 0.49) | | | | | | |
| **Total (95% CI)** | | **7333** | | **7129** | **100.0%** | **0.99 [0.94, 1.05]** |
| Total events | 1806 | | 1775 | | | |
| Heterogeneity: $Tau^2 = 0.00$; $Chi^2 = 8.42$, df = 9 (P = 0.49); $I^2 = 0\%$ | | | | | | |
| Test for overall effect: Z = 0.31 (P = 0.75) | | | | | | |
| Test for subgroup differences: $Chi^2 = 1.09$, df = 1 (P = 0.30), $I^2 = 8.2\%$ | | | | | | |

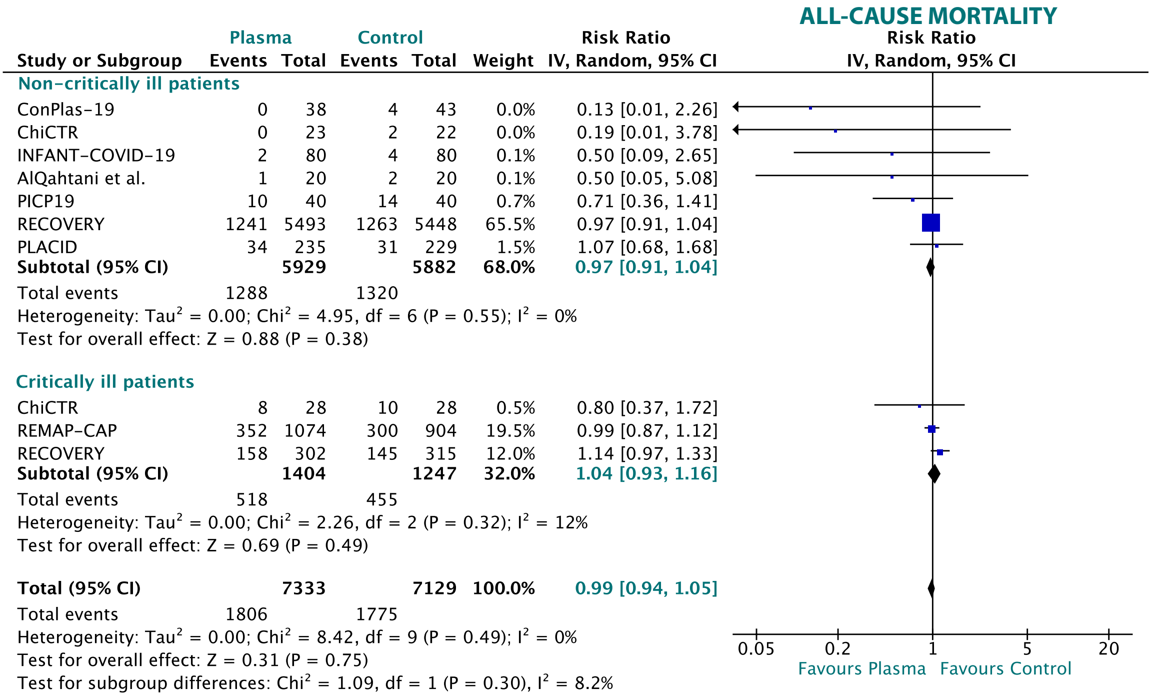


**Appendix Figure 2** Forrest plot depicting the risk ratio of all-cause mortality between convalescent plasma and control in the subgroups of non-critically and critically ill patients.

**ALL-CAUSE MORTALITY**
**by anti-SARS-CoV-2 antibody status**

| Study or Subgroup | Plasma Events | Plasma Total | Control Events | Control Total | Weight | Risk Ratio IV, Random, 95% CI |
|---|---|---|---|---|---|---|
| **Anti-SARS-CoV-2 antibody status negative** | | | | | | |
| REMAP-CAP | 130 | 270 | 78 | 148 | 8.1% | 0.91 [0.75, 1.11] |
| RECOVERY | 642 | 2016 | 558 | 1660 | 36.0% | 0.95 [0.86, 1.04] |
| **Subtotal (95% CI)** | | **2286** | | **1808** | **44.1%** | **0.94 [0.87, 1.02]** |
| Total events | 772 | | 636 | | | |
| Heterogeneity: Tau² = 0.00; Chi² = 0.11, df = 1 (P = 0.74); I² = 0% | | | | | | |
| Test for overall effect: Z = 1.42 (P = 0.16) | | | | | | |
| **Anti-SARS-CoV-2 antibody status positive** | | | | | | |
| REMAP-CAP | 190 | 597 | 135 | 409 | 9.5% | 0.96 [0.80, 1.16] |
| RECOVERY | 575 | 3078 | 501 | 2810 | 26.6% | 1.05 [0.94, 1.17] |
| **Subtotal (95% CI)** | | **3675** | | **3219** | **36.1%** | **1.03 [0.93, 1.12]** |
| Total events | 765 | | 636 | | | |
| Heterogeneity: Tau² = 0.00; Chi² = 0.60, df = 1 (P = 0.44); I² = 0% | | | | | | |
| Test for overall effect: Z = 0.52 (P = 0.60) | | | | | | |
| **Anti-SARS-CoV-2 antibody status unknown** | | | | | | |
| RECOVERY | 182 | 701 | 349 | 1293 | 13.2% | 0.96 [0.82, 1.12] |
| REMAP-CAP | 81 | 205 | 132 | 348 | 6.7% | 1.04 [0.84, 1.29] |
| **Subtotal (95% CI)** | | **906** | | **1641** | **19.8%** | **0.99 [0.87, 1.12]** |
| Total events | 263 | | 481 | | | |
| Heterogeneity: Tau² = 0.00; Chi² = 0.35, df = 1 (P = 0.56); I² = 0% | | | | | | |
| Test for overall effect: Z = 0.19 (P = 0.85) | | | | | | |
| **Total (95% CI)** | | **6867** | | **6668** | **100.0%** | **0.98 [0.93, 1.04]** |
| Total events | 1800 | | 1753 | | | |
| Heterogeneity: Tau² = 0.00; Chi² = 2.86, df = 5 (P = 0.72); I² = 0% | | | | | | |
| Test for overall effect: Z = 0.71 (P = 0.48) | | | | | | |
| Test for subgroup differences: Chi² = 1.81, df = 2 (P = 0.40), I² = 0% | | | | | | |

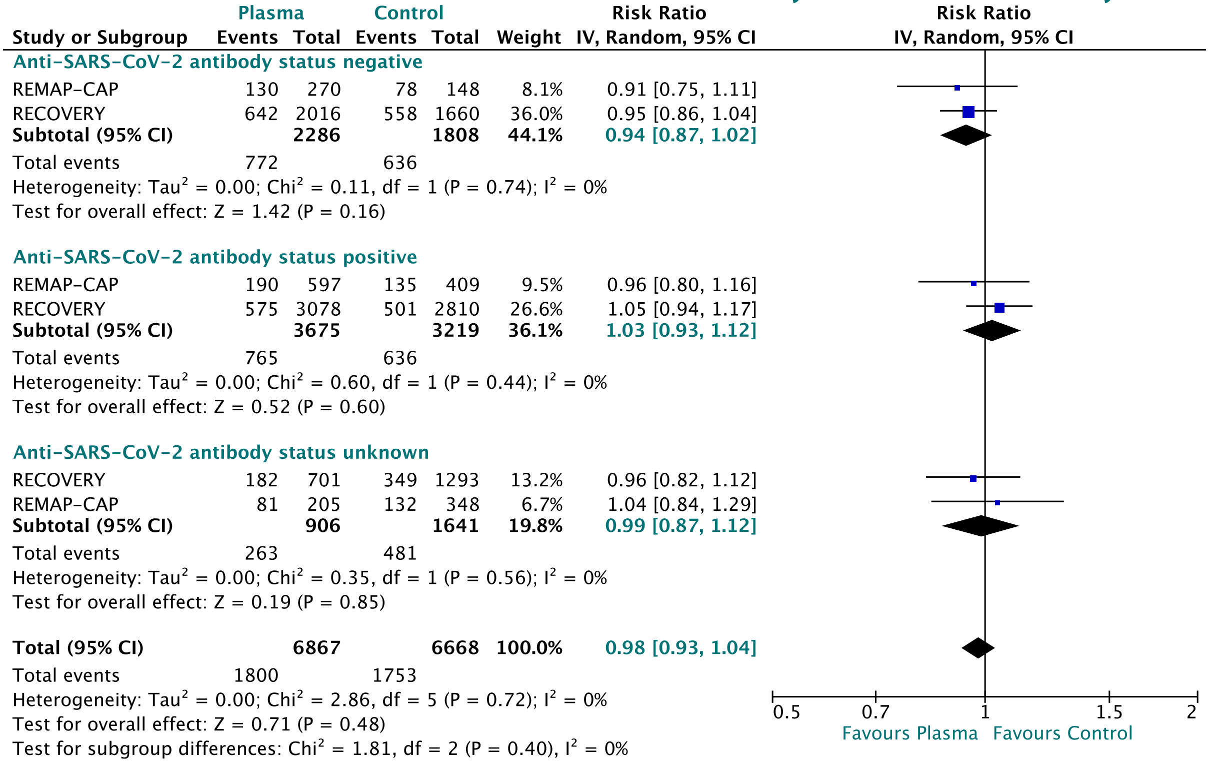


**Appendix Figure 3** Forrest plot depicting the risk ratio of all-cause mortality between convalescent plasma and control in the subgroups of patients with and without preexisting anti-SARS-CoV-2 antibodies at baseline.

## MECHANICAL VENTILATION USE

| Study | Plasma Events | Plasma Total | Control Events | Control Total | Weight | Risk Ratio IV, Random, 95% CI |
|---|---|---|---|---|---|---|
| **Studies published in peer-reviewed journals** | | | | | | |
| INFANT-COVID-19 | 2 | 80 | 4 | 80 | 0.3% | 0.50 [0.09, 2.65] |
| AlQahtani et al. | 4 | 20 | 6 | 20 | 0.8% | 0.67 [0.22, 2.01] |
| PlasmAr | 19 | 228 | 10 | 105 | 1.7% | 0.88 [0.42, 1.82] |
| RECOVERY | 678 | 5493 | 690 | 5448 | 93.8% | 0.97 [0.88, 1.08] |
| PLACID | 19 | 227 | 19 | 224 | 2.5% | 0.99 [0.54, 1.81] |
| O'Donnell et al. | 12 | 150 | 4 | 73 | 0.8% | 1.46 [0.49, 4.37] |
| **Subtotal (95% CI)** | | **6198** | | **5950** | **99.9%** | **0.97 [0.88, 1.07]** |
| Total events | 734 | | 733 | | | |
| Heterogeneity: $Tau^2 = 0.00; Chi^2 = 1.67, df = 5 (P = 0.89); I^2 = 0\%$ | | | | | | |
| Test for overall effect: Z = 0.60 (P = 0.55) | | | | | | |
| **Studies published as preprints** | | | | | | |
| ConPlas-19 | 0 | 38 | 1 | 43 | 0.1% | 0.38 [0.02, 8.97] |
| **Subtotal (95% CI)** | | **38** | | **43** | **0.1%** | **0.38 [0.02, 8.97]** |
| Total events | 0 | | 1 | | | |
| Heterogeneity: Not applicable | | | | | | |
| Test for overall effect: Z = 0.60 (P = 0.55) | | | | | | |
| **Total (95% CI)** | | **6236** | | **5993** | **100.0%** | **0.97 [0.88, 1.07]** |
| Total events | 734 | | 734 | | | |
| Heterogeneity: $Tau^2 = 0.00; Chi^2 = 2.02, df = 6 (P = 0.92); I^2 = 0\%$ | | | | | | |
| Test for overall effect: Z = 0.62 (P = 0.54) | | | | | | |
| Test for subgroup differences: $Chi^2 = 0.34, df = 1 (P = 0.56), I^2 = 0\%$ | | | | | | |

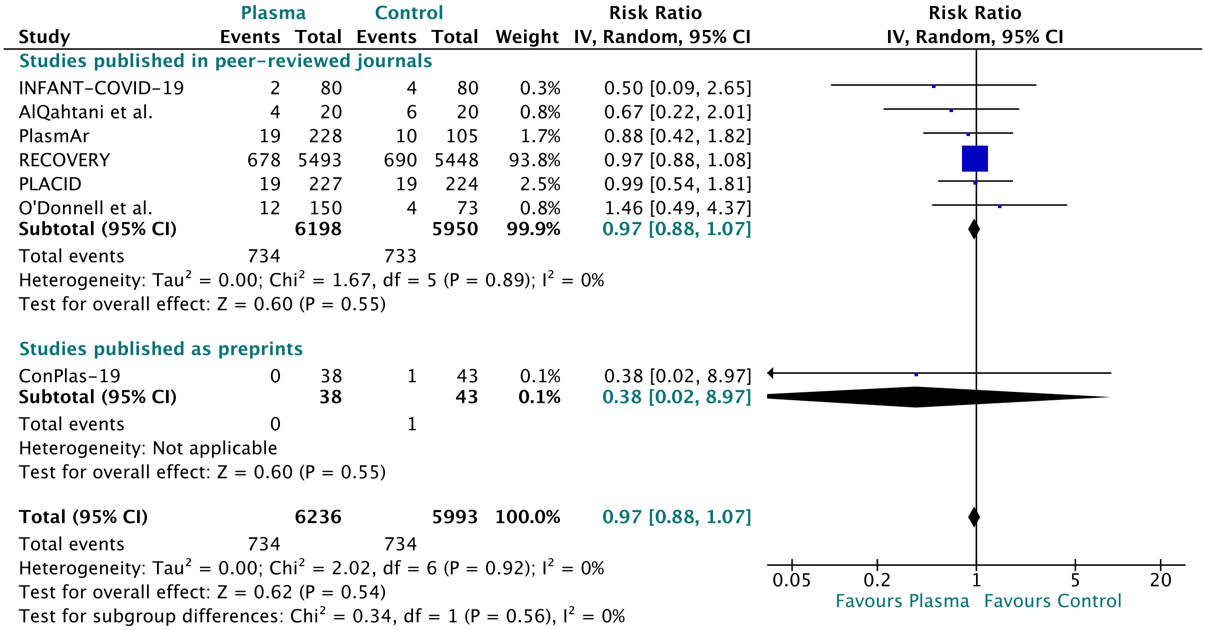


**Appendix Figure 4** Forrest plot depicting the risk ratio of requirement of mechanical ventilation after enrollment between convalescent plasma and control.

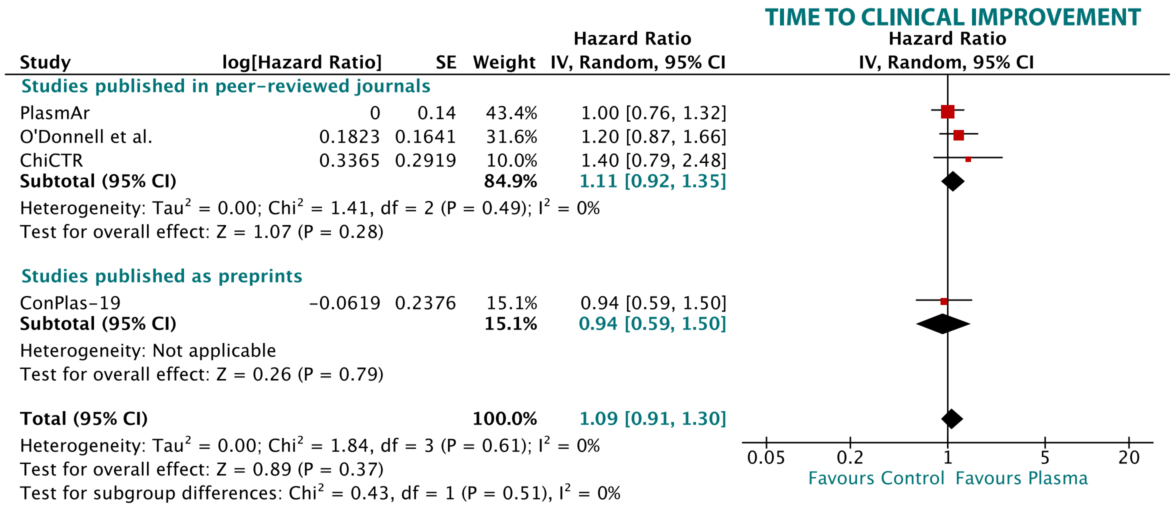


**Appendix Figure 5** Forrest plot depicting the hazard ratio for the time to clinical improvement between convalescent plasma and control.

TIME TO HOSPITAL DISCHARGE

| Study | log[Hazard Ratio] | SE | Weight | Hazard Ratio IV, Random, 95% CI |
|---|---|---|---|---|
| **Studies published in peer-reviewed journals** | | | | |
| Pouladzadeh et al. | −0.9997 | 1.4859 | 0.1% | 0.37 [0.02, 6.77] |
| ConCOVID | −0.1278 | 0.2987 | 1.5% | 0.88 [0.49, 1.58] |
| CONCOR-1 | −0.0943 | 0.0657 | 30.9% | 0.91 [0.80, 1.04] |
| REMAP-CAP | −0.0513 | 0.0508 | 51.6% | 0.95 [0.86, 1.05] |
| PlasmAr | 0 | 0.14 | 6.8% | 1.00 [0.76, 1.32] |
| O'Donnell et al. | 0.0198 | 0.1569 | 5.4% | 1.02 [0.75, 1.39] |
| ChiCTR | 0.4762 | 0.3082 | 1.4% | 1.61 [0.88, 2.95] |
| **Subtotal (95% CI)** | | | **97.6%** | **0.95 [0.88, 1.02]** |
| Heterogeneity: Tau² = 0.00; Chi² = 4.17, df = 6 (P = 0.65); I² = 0% | | | | |
| Test for overall effect: Z = 1.40 (P = 0.16) | | | | |
| **Studies published as preprints** | | | | |
| ConPlas-19 | 0.1222 | 0.2371 | 2.4% | 1.13 [0.71, 1.80] |
| **Subtotal (95% CI)** | | | **2.4%** | **1.13 [0.71, 1.80]** |
| Heterogeneity: Not applicable | | | | |
| Test for overall effect: Z = 0.52 (P = 0.61) | | | | |
| **Total (95% CI)** | | | **100.0%** | **0.95 [0.89, 1.02]** |
| Heterogeneity: Tau² = 0.00; Chi² = 4.69, df = 7 (P = 0.70); I² = 0% | | | | |
| Test for overall effect: Z = 1.30 (P = 0.19) | | | | |
| Test for subgroup differences: Chi² = 0.52, df = 1 (P = 0.47), I² = 0% | | | | |

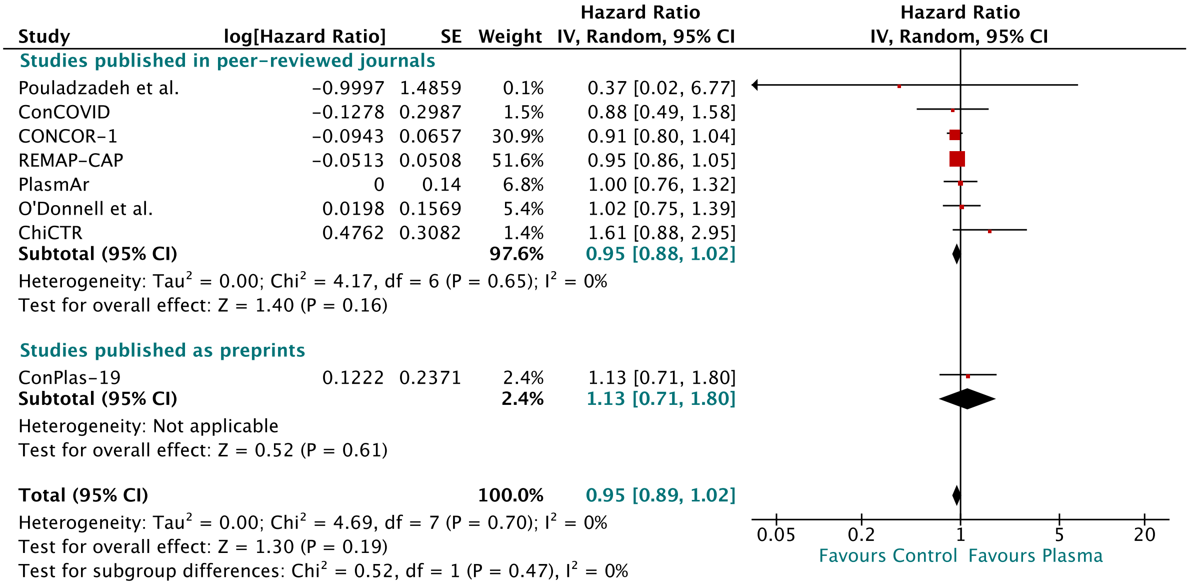


**Appendix Figure 6** Forrest plot depicting the hazard ratio for the time to hospital discharge between convalescent plasma and control.
